# Rapidly progressive Kaposi’s Sarcoma in an Iraqi boy received Valproic acid: a case report and review of literature

**DOI:** 10.1186/s12887-016-0653-3

**Published:** 2016-07-26

**Authors:** Lika’a Fasih Y. Al-Kzayer, Peter Keizer, Farah T. Abdulraheem, Kenji Sano, Minoru Kamata, Kazuo Sakashita, Laith A. Y. Habbaba, Kenichi Koike

**Affiliations:** 1 Department of Paediatrics, Shinshu University School of Medicine, 3-1-1, Asahi, Matsumoto, Nagano 390-8621 Japan; 2 Department of Emergency Medicine, Dr. G. B. Cross Memorial Hospital, Eastern Health, Clarenville, Newfoundland and Labrador, Canada; 3 Department of Laboratory, Al-Hamdaniya General Hospital, Mosul, Iraq; 4 Department of Laboratory Medicine, Shinshu University Hospital, Matsumoto, Nagano Japan; 5 Japan Chernobyl Foundation (JCF) NPO, Matsumoto, Nagano Japan; 6 Nagano Children’s Hospital, Hematology/Oncology, Matsumoto, Nagano Japan; 7 Department of Orthopaedic Surgery, Al-Hamdaniya General Hospital, Mosul, Iraq

**Keywords:** Kaposi’s sarcoma (KS), Classic Kaposi sarcoma (CKS), Valproic acid (VPA), Human herpesvirus-8 (HHV-8), Cerebral palsy (CP)

## Abstract

**Background:**

Kaposi’s sarcoma (KS), an endothelial neoplasm, is associated with human herpes virus (HHV) -8 infection. KS has four clinical sub-types: Mediterranean/classic, African/endemic, human immunodeficiency virus (HIV) -associated/epidemic, and transplantation-related/iatrogenic. Immunosuppression is an important cofactor in KS process. Classic KS (CKS) is exceedingly rare in children and when occurs, it is much more disseminated than adults. The epidemic, HIV-associated and the iatrogenic forms of childhood KS are a result of a profound and acquired T-cell deficiency. To our knowledge, this is the first paediatric KS case report from Iraq. Our patient was showing an unusual aggressive course of the disease while receiving Valproic acid (VPA) of the potential immune-suppressive effect.

**Case presentation:**

A six-year-old Iraqi boy, who had cerebral palsy (CP) and epilepsy since the age of 9-months, had received VPA to control his seizures. He developed skin discoloration followed by nodules that disseminated proximally from the lower extremities to the groin, face, ears and oral cavity, and then he died from severe respiratory distress after 110 days from the disease evolution. KS diagnosis was proved by a skin biopsy. As the patient was of Arab-Asian ethnicity and was HIV-seronegative status, accordingly, his condition best fitted the classic form of KS. However, recent studies showed the link of VPA with the reactivation of HHV-8. Moreover, accumulated experimental and clinical data elucidated that VPA induces T-cell suppression. Given that there was a lack of facilities to perform the laboratory immunological diagnostic tests in Iraq, the VPA-induced effect on immunity in our case (iatrogenic KS) could not be evaluated.

**Conclusions:**

Our report demonstrates a rare, rapidly progressing paediatric KS case and highlights the possible role of the 5-years’ administration of VPA and its challenging effect on cellular immunity based on recent studies. Thus, VPA could have promoted the development of the KS in our patient. This report also recalls the need of paediatricians to consider KS especially when the skin lesion appears at the child’s foot even in countries outside the geographical map of the disease.

**Electronic supplementary material:**

The online version of this article (doi:10.1186/s12887-016-0653-3) contains supplementary material, which is available to authorized users.

## Background

Kaposi’s sarcoma (KS), an endothelial neoplasm, was first reported by Moritz Kaposi in 1872 [1]. Chang et al., in 1994, isolated KS-associated herpesvirus, also known as human herpesvirus (HHV) -8, from KS lesion [2]. KS has four clinical sub-types: Mediterranean/classic, African/endemic, human immunodeficiency virus (HIV) -associated/epidemic, and transplantation-related/iatrogenic [3, 4]. Classic KS (CKS) is generally an indolent disease involving the dermis of the lower extremities, and affects predominantly elderly men of Mediterranean, Eastern European, and Jewish heritage, but it is exceedingly rare in children [1, 5]. Worldwide occasional cases of paediatric CKS have been reported, revealing a more disseminated form than adult CKS [6]. Moreover, the course may be more rapid when the viscera are involved, due not to greater aggressiveness, but to complications including haemorrhage [7, 8]. In advanced cases, the lesions may spread to ears, fingers, and mucous membranes. Lymph nodes and/or visceral involvement may subsequently occur [9]. Pulmonary involvement in KS occurs commonly in immunosuppressed patients, who usually have had preceding mucocutaneous involvement [10–12]. African/endemic KS follows a more severe course and is common in Sub-Saharan Africa, it affects younger adults with a rapidly progressive lymphadenopathic course [4]. Epidemic and Iatrogenic KS, which generally show the most severe courses, remain relatively rare in children [5, 10, 13]. The iatrogenic type of KS, also called immunosuppression-associated KS, can be triggered by medical treatment using immunosuppressant medications, for various reasons including post-organ transplantation. The epidemic and iatrogenic forms of childhood KS result from a profound and acquired T cell deficiency [14].

Of note, KS is >100 times more common in immunosuppressed individuals. Loss of T-cell-mediated immune control allows HHV-8-infected cells to proliferate unchecked and thus the tumours to develop [15].

The prevalence of HHV-8 varies geographically; with a seropositivity level exceeds 40 % in parts of Africa and South America, ranges from 30 %–40 % in the Mediterranean Basin, and drops to 20 % in non-endemic areas such as North America, Northern Europe, and most of Asia. However, the vast majority of HHV-8 infected individuals (more than 40 % of individuals in some populations, based on seroprevalence) do not develop KS. The epidemiology of HHV-8 infection and development of iatrogenic KS in the paediatric population has not been fully elucidated yet [14]. Unfortunately, due to lack of diagnostic facilities, information is not yet available in regard to the epidemiology of HHV-8 in Iraq.

The following is a paediatric KS case from Iraq who was showing an unusual aggressive course of the disease while receiving Valproic acid (VPA) of the potential immune-suppressive effect. To our knowledge this is the first paediatric KS case report from Iraq.

## Case presentation

A 6-year-old Iraqi boy of Arab-Asian ethnicity who had developed cerebral palsy (CP) with mental retardation and epilepsy after encephalitis at the age of 9 months. Since that time, he had been treated with oral VPA (30 mg/kg/day, twice a day) to control the seizures. His family history was unremarkable apart from that his next older brother had acute lymphoblastic leukaemia and died due to severe infection a year and a half before the birth of this case of KS. Our patient was the youngest among 4 siblings of healthy second-cousin parents. The other 2 older siblings are healthy. Our patient suffered from recurrent respiratory tract infections and was hospitalized twice. The latest episode was on December 1st, 2013 when he was 6 years-old. Although he had middle lobe atelectasis, he responded well to a 1-week treatment with non-specific antibiotic and his chest roentgenogram showed improvement.

**Fig. 1:**
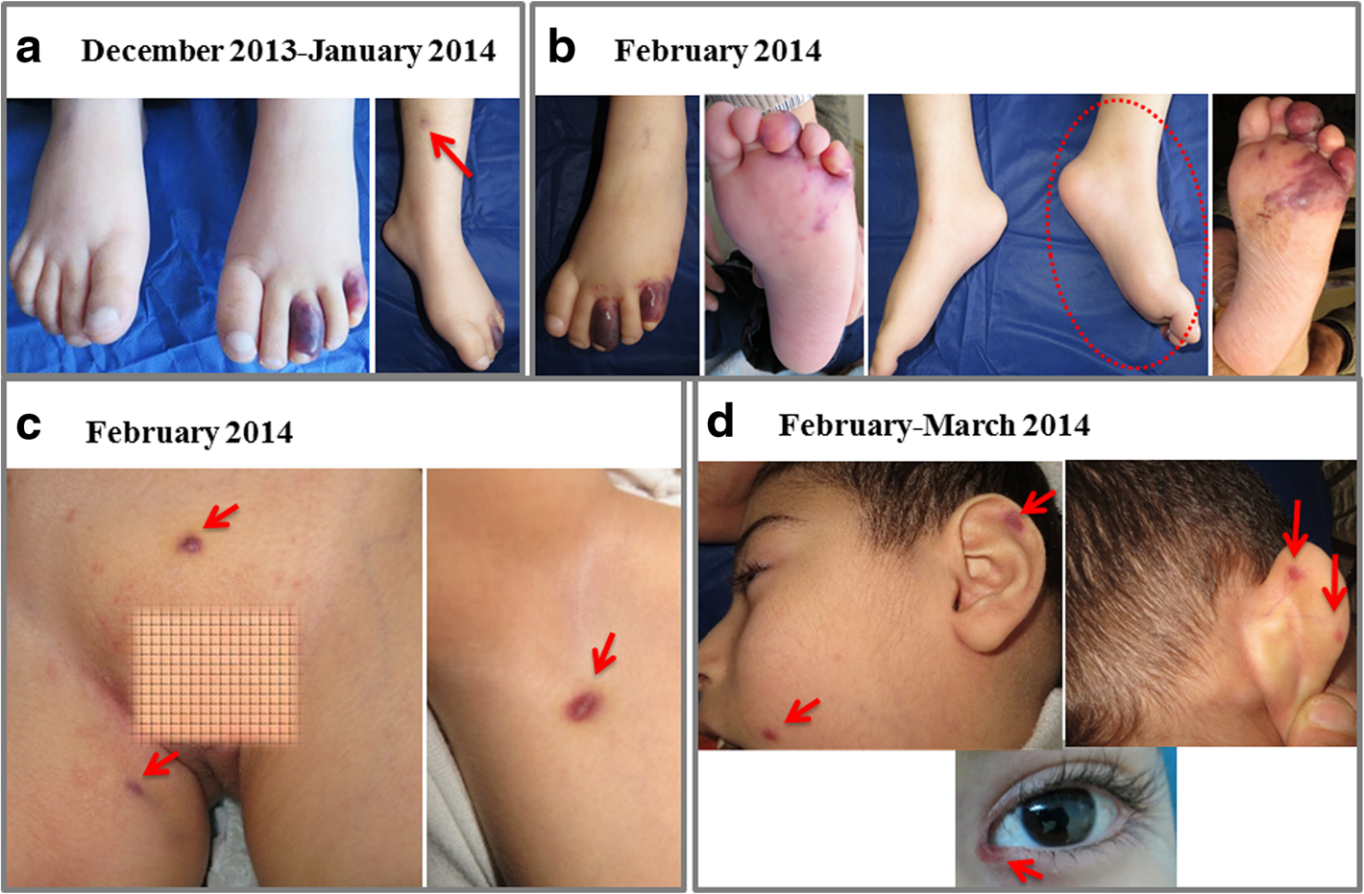
Clinical progress of the patient. Skin discoloration of KS on the toes of the left foot, then a nodule appeared on *left* leg arrowed (**a**). The lesion extended to plantar surface with *left* leg edema encircled (**b**). New nodules appeared at the groin and right axilla (indicated by *arrows*) (**c**). KS nodules appeared on face, ears, and the angle of the left eye (indicated by *arrows*) (**d**)

One week after being discharged from the hospital, painless skin lesions appeared in the form of a bluish-purple plaque (blue-violet to black hue) on the middle and little toes of his left foot and extended to the upper lateral sole area (Fig. 1a). Physical examination revealed a spastic quadriplegic type of CP, mentally subnormal with open mouth, mouth breathing, and misalignment of the teeth with malocclusion. He was in full-body hypertonia with legs being affected more than arms and hyperreflexia with clonus. Systemic examination of the chest revealed harsh vesicular breath sound and normal heart sounds. His abdomen was soft, with no organomegaly, mass or free fluid. The investigations performed included complete blood count, blood film, erythrocyte sedimentation rate, coagulation profile, blood chemistry, liver and renal work-up, urinalysis, chest x-ray and ultrasound examination of the abdomen were all normal. Serological screening for hepatitis B and C as well as HIV was negative.

In January 2014, palpable bluish-purple nodules appeared on the left leg and then on the right leg. The skin discoloration extended to the plantar surface, and edema of the left foot became obvious (Fig. 1b), but peripheral arterial pulse and the perfusion in the lower extremities were intact. A skin biopsy was taken on February 15th, 2014 from the lesion on the left foot, thereafter the patient experienced pain. During February and March 2014, new skin nodules appeared in an ascending pattern: 2 around the groin, 2 on the left arm, 1 in the right sub-axillary area, and on the face (medial angle of left eye and both ear cartilages) (Fig. 1c, d). Small cervical lymphadenopathy also became palpable. Meanwhile, a purple, slightly elevated lesion erupted on the hard palate. He was kept on his VPA dose with no additional therapy apart from analgesic agents, as the family refused hospital admission. On April 3rd, 2014 the patient had developed sudden respiratory distress, and died before arrival to the hospital just 3 days before getting his final biopsy test results.

**Fig. 2:**
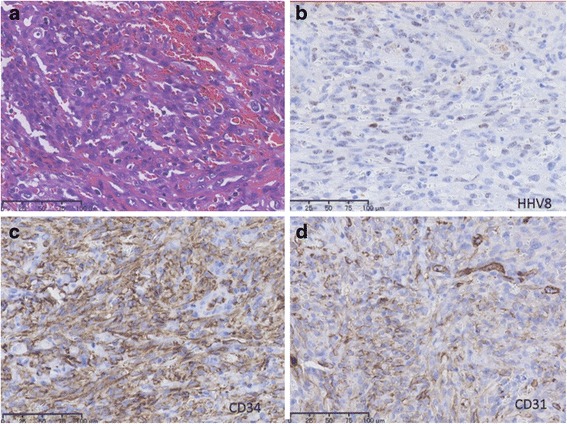
Skin biopsy findings. Spindle cells were closely associated with narrow vascular spaces, numerous erythrocytes and few inflammatory cells (H&E) (**a**). Some of the spindle cells showed nuclear expression of HHV-8 (**b**). Most of the spindle cells showed apparent expression of CD34 and CD31 (**c**, **d**)

The skin biopsy material was re-evaluated in Japan because of the lack of facilities for HHV-8 detection in Iraq. The results are presented in Fig. 2, the spindle cells reacted with monoclonal antibodies against CD34 and CD31 antigens. Additionally, these cells were positive for HHV-8, hence the diagnosis of KS was confirmed. The “CARE” checklist is available as Additional file 1. This child’s clinical course is summarized in the accompanying supplemental time-line file (Additional file 2).

## Discussion

Given the accessible published literatures since 1960 excluding African, iatrogenic and HIV-associated KS, there were 36 paediatric KS cases of 15 years of age or younger including our case were identified. Of them, 32 had skin lesions and 4 had no skin lesion [5–9, 11–13, 16–31]. Twenty-four (67 %) of 36 KS patients belonged to the Mediterranean Basin, including Turkey (8 cases) and Italy (7 cases) as shown in Table 1. Two-thirds of patients were under 10-years old, and there was a male gender predominance with 75 % being boys. Skin lesion was the first clinical presentation in 75 % of the cases, lymph nodes were palpable in 65 %, and visceral involvement was reported in more than half of the cases.

**Table 1:** Pediatric classic Kaposi’s sarcoma in literature review

Variable	KS with skin lesion		KS with no skin lesion		*Total*
	*number = 32*		*number = 4*		
Origin	Mediterranean Basin	Other countries	Mediterranean Basin	Other countries	
	22	10	2	2	*36*
Age					
0- < 10	11	7	1	2	*21*
10- < =15	8	3	1	0	*12*
NA	3		0	0	*3*
Sex					
Male	15	8	2	2	*27*
Female	6	1	0	0	*7*
NA	1	1	0	0	*2*
Clinical findings					
Lymph node (s)					
*Yes*	10	1	0	2	*13*
*No*	2	5	0	0	*7*
*NA*	10	4	2	0	*16*
Visceral involvement					
*Yes*	8	3	1	2	*14*
*No*	6	4	1	0	*11*
*NA*	8	3	0	0	*11*
Diagnosis					
Skin biopsy	18	8	0	0	*26*
Lymph node biopsy	1 ^a^	0	1	2	*3*
NA	4	2	1	0	*7*
HHV-8 status					
Positive	12	2	0	0	*14*
Negative	1 ^b^	0	0	0	*1*
Non-conclusive	1	0	0	0	*1*
Test not yet available (old reports)	8	8	2	2	*20*
Immunity status					
Identified Immunodeficiency	5	0	0	0	*5*
*1- IFN-gamma-R1 deficiency*	2	0	0	0	
*2- Wiskott-Aldrich syndrome*	2	0	0	0	
*3- Inherited human OX40 deficiency*	1	0	0	0	
Unidentified Immunodeficiency	2	2	0	0	*4*
NA	15	8	2	2	*27*
Treatment					
Given treatment					*21*
No treatment	1	1	0	0	*2*
NA	6	5	2	0	*13*
Outcome					
Survived	10	5	0	1	*16*
Dead	6	1	1	1	*9*
NA	6	4	1	0	*11*

*HHV-8* Human herpes virus-8, *IFN* Interferon, *KS* Kaposi’s sarcoma, *NA* not available

^a^One case with skin and lymph node biopsy

^b^(reference 18, 1999)

KS was diagnosed on the basis of skin biopsies demonstrating the presence of spindle cells in 90 % of cases. HIV was negative in all 26 examined cases, and HHV-8 was positive in 87.5 % of the examined cases. Within the collected series, 16 (64 %) of 25 cases with known outcome were reported as survivors, including two cases rescued by Hematopoietic stem cell transplantation, and five of the 16 survivors had coexisted identified immunodeficiency. The remaining 9 cases were reported dead, eight of them had visceral involvement and five of them died within < 6 months of the onset of the disease.

In our patient, KS was suspected by the evolution from a macular stage, with characteristic colour, multifocal distribution and the presence of lesions on the toes. The diagnosis was made by the characteristic skin biopsy findings along with immunohistochemical detection of HHV-8.

Regarding the risk factors in our patient, it is well known that the development of KS is a multifactorial process in which HHV-8 infection is a prerequisite and an immunosuppression is an important cofactor [12, 32, 33]. Recently, Gorres et al., reported the link of VPA with the reactivation of HHV-8 from latency. Thus, the virus could persist and spread among hosts’ cells, and therefore could play a role in carcinogenesis [34]. Accumulated experimental and clinical data also showed that VPA induces T-cell suppression via a direct effect and effectively reduces the expression of pro-inflammatory cytokines such as IFN-ɤ [35]. Therefore, a possible risk factor for developing KS might be the use of VPA in our case. However, given that there was a lack of facilities to perform laboratory immunological diagnostic tests in Iraq, the VPA-induced (iatrogenic) effect on immunity in our particular case could not be proved. Moreover, if we take into consideration his previous history of encephalitis, which might be of herpes simplex etiology and his repeated respiratory tract infections, then single-gene immunodeficiencies could not be excluded as a predisposing factor for KS according to literatures as well [14].

CKS has been reported in two isolated studies among Iraqi adults: a 21-patient report over a 10 year period (1974–1984) and a more aggressive 20-case report over a shorter period (1999–2001) with a predilection for younger age [36]. Taken together, the increased number of cases along with the aggressive behaviour of CKS in Iraq suggested a potential environmental factor. Depleted uranium (DU) has been used in Iraq since 1991 (the first Gulf War) contaminating the environment through its radioactive and toxic effects. A possible relationship between KS and DU has been suggested by Shelleh [37]. Since children are more susceptible to radiation than adults, a possible role of DU in the development of KS cannot be excluded in our case.

Finally, despite the scarcity of the KS and the shortage of the diagnostic tools in Iraq, diagnosis of KS was possible with multiple consultations and cooperation from inside and outside Iraq and then it was proved in Japan. In reference to KS subtype, our patient was of Arab-Asian ethnicity, and was HIV-seronegative, accordingly, his condition could be categorized as a classic form of KS; however, the iatrogenic VPA-induced-KS could not be excluded.

## Conclusion

Our report demonstrates one of the rare, rapidly progressing paediatric KS and highlights the possible role of the 5-years’ administration of VPA and its challenging effect on cellular immunity based on recent studies. Thus, VPA could have promoted the development of the KS in our patient. This report also recalls the need of paediatricians to consider KS especially when the skin lesion appears at the child’s foot even in countries outside the geographical map of the disease.

## Abbreviations

CKS, Classic Kaposi’s sarcoma; CP, cerebral palsy; DU, depleted uranium; HHV-8, human herpes virus −8; HIV, human immunodeficiency virus; IFN-ɤ, Interferon-gamma; KS, Kaposi’s sarcoma; VPA, valproic acid

## Additional files

Additional file 1: CARE checklist of information to include when writing a case report. (DOC 1547 kb) Additional file 2: Time-line table. Summarization of the child’s clinical course. (DOCX 48 kb)
