# Supplementary material for: Rapidly progressive Kaposi’s Sarcoma in an Iraqi boy received Valproic acid: a case report and review of literature
Source: BMC Pediatr. 2016 Jul 26;16:111. doi: 10.1186/s12887-016-0653-3 (PMC4962423; doi:10.1186/s12887-016-0653-3)
Supplement: Additional file 2: — Time-line table. Summarization of the child’s clinical course. (DOCX 48 kb) [file 12887_2016_653_MOESM2_ESM.docx]

| Age  (DOB): 17/7/2008 | Date | Clinical information | Management |
| --- | --- | --- | --- |
| 9-month | April/ 2009 | Encephalitis, Epilepsy, CP with mental retardation | VPA |
| 6-year | 1/12/2013 | Acute respiratory infection complicated by middle lobe atelectasis | Hospital admission, 1-week non-specific antibiotic & kept on VPA |
|  | 8/12/2013 | Improved clinically and radiologically | Discharged and kept on VPA |
|  | 13/12/2013 | painless skin lesions on left foot | VPA/ Pediatric clinic in the hospital/no treatment  Follow up was advised |
|  | Jan/2014 | Nodules on both legs | VPA/ consultation started for different departments |
|  | Feb/2014 | Oedema of left leg | VPA/ consultation and case presentation |
|  | 15/Feb/2014 | Skin biopsy | VPA/ 3 histopathologists in Iraq had discussed |
|  | Feb/2014 | Lesion extended to planter surface with pain | VPA/ analgesia |
|  | Feb-Mar /2014 | New skin nodules: groin/arm/axilla/face | VPA/ analgesia |
|  | Mar/2014 | Skin biopsy (consult) result: suspicion of KS in Iraq | VPA/ analgesia/ international consultations started |
|  | Mar/2014 | Cervical lymph node | VPA/ analgesia/ sample was send to Japan |
|  | Mar/2014 | Lesion in hard palate | VPA/ family refused hospitalization |
|  | 3/April/2014 | Sudden respiratory distress | VPA |
| Death : 3/April/2014 | | Died before arrival to hospital | |
| 6/April/2014 | | Diagnosis of KS and detection of HHV-8 was proved in Japan | |

**Time-line table: Summary of the child’s clinical course**

Born

1994

Alive

Healthy

Born 1990

Alive

Healthy

Our Index case

Born

2000

Developed

ALL in 2005

Hepatitis B+ve

Died 19/2/2007

Second-cousin

Born 1965

Alive

Healthy

Born 1965

Alive

Healthy

ALL
